# [Retracted] Functions of endothelin‑1 in apoptosis and migration in hepatocellular carcinoma

**DOI:** 10.3892/etm.2025.12841

**Published:** 2025-03-10

**Authors:** Lu Shi, Shan-Shan Zhou, Wan-Bo Chen, Lei Xu

Exp Ther Med 13:3116–3122, 2017; DOI: 10.3892/etm.2017.4314

Following the publication of this paper, it was drawn to the Editor’s attention by a concerned reader that certain of the flow cytometric assay data shown in Fig. 3D on p. 3120 were strikingly similar to data appearing in different form in another article written by different authors at different research institutes that had already been submitted for publication to the journal *Molecular Medicine Reports* (and which has subsequently been retracted), prior to the receipt of this article at *Experimental and Therapeutic Medicine*. In view of the fact that the abovementioned data had already already been submitted for publication before this article was received at *Experimental and Therapeutic Medicine*, the Editor has decided that this paper should be retracted from the Journal. The authors were asked for an explanation to account for these concerns, but the Editorial Office did not receive a reply. The Editor apologizes to the readership for any inconvenience caused.

